# CYP2D6 activity and the risk of recurrence of *Plasmodium vivax* malaria in the Brazilian Amazon: a prospective cohort study

**DOI:** 10.1186/s12936-017-2139-7

**Published:** 2018-02-01

**Authors:** Larissa W. Brasil, Fernanda Rodrigues-Soares, Ana B. Santoro, Anne C. G. Almeida, Andrea Kühn, Rajendranath Ramasawmy, Marcus V. G. Lacerda, Wuelton M. Monteiro, Guilherme Suarez-Kurtz

**Affiliations:** 1 Diretoria de Ensino e Pesquisa, Fundação de Medicina Tropical Dr. Heitor Vieira Dourado, Manaus, AM Brazil; 2 Programa de Pós-Graduação em Medicina Tropical, Universidade do Estado do Amazonas, Manaus, AM Brazil; 3 Coordenação de Pesquisa, Instituto Nacional de Câncer, Rio de Janeiro, RJ 20231-050 Brazil; 4 Faculdade de Medicina, Universidade Nilton Lins, Manaus, AM Brazil; 5 Instituto Leônidas & Maria Deane, Fiocruz, Manaus, AM Brazil

**Keywords:** *Plasmodium vivax*, Malaria recurrence, *CYP2D6* gene, Polymorphisms, CYP2D6 activity score, Primaquine, Pharmacogenomics

## Abstract

**Background:**

CYP2D6 pathway mediates the activation of primaquine into active metabolite(s) in hepatocytes. *CYP2D6* is highly polymorphic, encoding CYP2D6 isoforms with normal, reduced, null or increased activity. It is hypothesized that *Plasmodium vivax* malaria patients with defective *CYP2D6* function would be at increased risk for primaquine failure to prevent recurrence. The aim of this study was to investigate the association of *CYP2D6* polymorphisms and inferred CYP2D6 phenotypes with malaria recurrence in patients from the Western Brazilian Amazon, following chloroquine/primaquine combined therapy.

**Methods:**

The prospective cohort consisted of *P. vivax* malaria patients who were followed for 6 months after completion of the chloroquine/primaquine therapy. Recurrence was defined as one or more malaria episodes, 28–180 days after the initial episode. Genotyping for nine *CYP2D6* SNPs and copy number variation was performed using TaqMan assays in a Fast 7500 Real-Time System. *CYP2D6* star alleles (haplotypes), diplotypes and CYP2D6 phenotypes were inferred, and the activity score system was used to define the functionality of the *CYP2D6* diplotypes. *CYP2D6* activity scores (AS) were dichotomized at ≤ 1 (gPM, gIM and gNM-S phenotypes) and ≥ 1.5 (gNM-F and gUM phenotypes).

**Results:**

Genotyping was successfully performed in 190 patients (44 with recurrence and 146 without recurrences). Recurrence incidence was higher in individuals presenting reduced activity CYP2D6 phenotypes (adjusted relative risk = 1.89, 95% CI 1.01–3.70; *p* = 0.049). Attributable risk and population attributable fraction were 11.5 and 9.9%, respectively. The time elapsed from the first *P. vivax* malaria episode until the recurrence did not differ between patients with AS of ≤ 1 versus ≥ 1.5 (*p* = 0.917).

**Conclusions:**

The results suggest that *CYP2D6* polymorphisms are associated with increased risk of recurrence of vivax malaria, following chloroquine–primaquine combined therapy. This association is interpreted as the result of reduced conversion of primaquine into its active metabolites in patients with reduced *CYP2D6* enzymatic activity.

## Background

Malaria is a global infectious disease, with 95 countries reporting transmission in 2015 and hundreds of millions of anti-malarial chemotherapies administered every year [1]. Anti-malarial drugs display considerable interindividual variability in disposition and clinical effects, whether beneficial or adverse, and there is evidence that genetic polymorphisms impacting drug pharmacokinetics and/or pharmacodynamics contribute to this variability [2–6]. Regarding *Plasmodium vivax* malaria, which prevails in the Brazilian Amazon where the present study was conducted, the World Health Organization (WHO) and the Brazilian Ministry of Health recommend combined chloroquine/primaquine as first-line therapy for uncomplicated disease in adults and children.

Primaquine is the only anti-malarial available clinically for prevention of *P. vivax* malaria relapses, which are classically associated with hypnozoite activation. From a pharmacogenomics perspective, the observation of primaquine-induced haemolysis in patients with G6PD deficiency [7] is not only a historical hallmark but a distinct example of clinical relevance of pharmacogenomics-informed c deficiency [7]. G6PD deficiency prevalence was estimated in 4.5% in the Western Brazilian Amazon and was related to decreased malaria susceptibility [8], which led us to develop studies for the implementation of a rapid test to detect the G6PD deficiency in patients diagnosed with malaria. Comparatively, the impact of other pharmacogenomic polymorphisms in the response and/or disposition of primaquine and chloroquine in *P. vivax* malaria patients has drawn less attention [9]. However, an original observation by Bennet et al. [10] followed by a case report by Ingram et al. [11] triggered considerable interest in a novel aspect of the pharmacogenomics of *P. vivax* malaria. These reports described relapses of *P. vivax* following chloroquine/primaquine combined therapy in three individuals with *CYP2D6* genotypes encoding *CYP2D6* isoforms with reduced (intermediate metabolizers) or null function (poor metabolizers). Relapses were ascribed to failure of primaquine hypnozoiticidal activity, which requires metabolic conversion of primaquine, a pro-drug, into redox-active metabolite(s) [5].

Evidence from metabolomic studies in vitro [12] and animal models in vivo [13] indicate that the *CYP2D6* pathway is largely responsible for primaquine activation in hepatocytes. Accordingly, *P. vivax* malaria patients with defective CYP2D6 function would be at increased risk for primaquine failure to prevent relapses [10, 11]. Indeed, St Jean et al. [14] verified a higher rate of relapse in intermediate CYP2D6 metabolizers, then in normal metabolizers in *P. vivax* patients from Peru and Thailand, and Silvino et al. [15] reported higher frequency of a *CYP2D6* SNP (rs1065852) in multiple-relapse versus single-relapse patients from the Central-West region of Brazil.

The aim of this study was to investigate the impact of *CYP2D6* polymorphisms and inferred CYP2D6 phenotypes on the risk of recurrence of *P. vivax* malaria in patients from the Brazilian Amazon, after completion of chloroquine/primaquine combined therapy.

## Methods

### Study cohort

A total of 213 patients living in a previously described community [16], located in the peri-urban area of Manaus, the capital city of the Amazonas state, were enrolled. All patients presented *P. vivax* malaria diagnosed by microscopy and confirmed by PCR [17] and were treated with the therapeutic scheme recommended by the Brazilian Ministry of Health for *P. vivax* uncomplicated malaria in children and adults, which consists of association of chloroquine (150 mg/day, 3 days) and primaquine (15 mg/day, 7 day) [18]. Treatment was not supervised. Consanguineous individuals, pregnant women and children with less than 6 months of age were excluded from the study. The present analyses are based on data from 190 patients, who were successfully genotyped for *CYP2D6* polymorphisms and copy number variation, and were followed up for 6 months after treatment, with monthly visits for blood sample collection and interviewing. *P. vivax* malaria recurrence was defined as one or more malaria episodes, 28–180 days after the initial episode.

### Laboratory procedures

Genomic DNA was isolated from peripheral blood by standard procedures, using the QIAamp^®^ DNA Mini Kit (QIAGEN^®^, Germany). *CYP2D6* genotyping was performed as described previously [19], using a Fast 7500 Real-Time System (Applied Biosystems, Foster City, CA). TaqMan assays were used following the manufacturer’s protocols for allele discrimination at nine *CYP2D6* polymorphic loci (Table 1) and for identification of *CYP2D6* gene deletion and duplication/multiplication.

**Table 1:** *CYP2D6* variants genotyped

Variant	ID SNP
− 1584C>G	rs1080985
31G>A	rs769258
100C>T	rs1065852
1023C>T	rs28371706
1846G>A	rs3892097
2615–2617delAAG	rs28371720
2988G>A	rs28371725
3183G>A	rs59421388
4180G>C	rs1135840

### *CYP2D6* alleles and diplotypes

*CYP2D6* diplotypes were inferred using HaploStats software (version 1.7.7) implemented on the R platform. Software-generated haplotypes were compared to the Human Cytochrome P450 (CYP) Allele Nomenclature Database [20], for star (*) allele designation. The CYP2D6*1 allele was set when no nucleotide change was observed in all genotyped SNPs. Haplotypes not matched with known *CYP2D6* alleles were grouped into the “other” category [19].

### Diplotype activity score and CYP2D6 phenotype

The activity score (AS) system [21, 22] was used to define the perceived functionality of the *CYP2D6* diplotypes. Briefly, values of 0-2 were assigned to the alleles identified in the study cohort, as follows: zero, no-function alleles (*4, *4xN, *5); 0.5, decreased-function (*9, *10, *17, *29, *41); 1, normal-function (*1, *2, *39) and 2, increased-function (*1xN, *2xN). The AS of diplotypes resulted from the sum of the assigned value to each allele. Patients with AS = 0, AS = 0.5, and AS > 2 were designated as genetic poor, intermediate, and ultrarapid metabolizers (gPM, gIM, and gUM), respectively. Patients with AS = 1, AS = 1.5, and AS = 2 were designated as genetic normal metabolizers (gNM), and sub-divided into gNM-slow (gNM-S, AS = 1) and gNM-fast (gNM-F, AS = 1.5–2).

### Statistical analysis

Allele and genotype frequencies of *CYP2D6* polymorphisms were derived by gene counting. Deviations from Hardy–Weinberg equilibrium were assessed by the goodness-of-fit Chi square test. Chi square tests were also applied to compare frequencies of minor alleles at the nine polymorphic loci genotyped, star alleles, inferred phenotypes and activity scores. Data from Friedrich et al. [19] for healthy individuals from the Brazilian North region, which encompasses the Brazilian Amazon, were used for power calculations regarding *CYP2D6* activity scores. A total of 45 patients with recurrence and 158 patients without recurrence confer a statistical power of 0.8, at a one-sided α level of 0.05 to detect a relative risk of 2.5 for the combined frequency of activity scores ranging from 0 to 1 (gPM, gIM and gNM-S phenotypes, see above) versus ≥ 1.5 (gNM-F and gUM). The relative risk (RR) of malaria recurrence among *CYP2D6* phenotypes was assessed by univariate analysis, adjusting by age and sex using a logistic regression. A Hosmer–Lemeshow test was used to validate the models used. Attributable risk (AR) and population attributable fraction (PAF) were also calculated. A Kaplan–Meier survival analysis was performed in order to detect differences in the time elapsed from the baseline malaria episode to the first recurrence between patients with null (gPM) or reduced (gIM or gNM-S) CYP2D6 metabolic activity compared to gNM-F or gUM metabolizers. Log-rank test was used to test differences according *CYP2D6* scores. Statistical significance was considered if *p* < 0.05. Analysis was made as described in the Stata software manual.

## Results

### Population characteristics

Of the 213 individuals recruited to the study, 190 (44 with recurrence and 146 without recurrence) were successfully genotyped for *CYP2D6* SNPs and copy number variation, and were included in the final analysis. Gender and age distribution were similar in both groups. Most of the patients presented only one recurrence episode (77.3%). Recurrences were observed more frequently 60–180 days after the first malaria episode (68.2%), with a mean time of 104 days (± 40.7; CI 95% 92.9–115.0) after the first episode. *CYP2D6* activity score ≤ 1 was 17.1% in the group with recurrence and 27.3% in the group without recurrence (Table 2).

**Table 2:** Demographic and clinical characteristics of the individuals included in the final analysis

Variable	Total (n, %)	Recurrence	
		No (n, %)	Yes (n, %)
Total	190 (100.0)	146 (76.8)	44 (23.2)
Gender (%)			
Male	107 (56.3)	84 (57.5)	23 (52.3)
Age (in years; %)			
0–18	81 (42.6)	62 (42.5)	19 (43.2)
19–60	98 (51.6)	75 (51.4)	23 (52.3)
> 60	11 (5.8)	9 (6.1)	2 (4.5)
Malaria recurrence			
1 episode	34 (17.9)	–	34 (77.3)
2 episodes	10 (5.3)	–	10 (22.7)
Time to first recurrence (in days)			
29–60	14 (7.4)	–	14 (31.8)
60–180	30 (15.8)	–	30 (68.2)
*CYP2D6* activity score			
≤ 1^a^	37 (19.5)	25 (17.1)	12 (27.3)
≥ 1.5^b^	153 (80.5)	121(82.9)	32 (72.7)

^a^Poor (gPM), intermediate (gIM) and normal slow (gNM-S) metabolizers

^b^Ultrarapid (gUM) and normal fast (gNM-F) metabolizers

### Distribution of *CYP2D6* polymorphisms and activity scores

The minor allele frequency (MAF) at the nine *CYP2D6* genotyped loci and the frequency distribution of the inferred *CYP2D6* star alleles in the overall study cohort are presented in Tables 3 and 4, respectively, along with data for healthy individuals from the Brazilian North region [19, 23].

**Table 3:** Minor allele frequency of *CYP2D6* polymorphisms

Variant	Friedrich et al. [19] (n = 474)	Current study		
		Total (n = 380)	Recurrence (n = 88)	No recurrence (n = 292)
− 1584C>G	0.229	0.203	0.115	0.229
31G>A	0.024	0.005	0.00	0.006
100C>T	0.142	0.197	0.214	0.192
1023C>T	0.059	0.029	0.049	0.024
1846G>A	0.128	0.086	0.088	0.085
2615–2617delAAG	0.010	0.009	0.000	0.012
2988G>A	0.040	0.059	0.069	0.057
3183G>A	0.024	0.011	0.000	0.015
4180G>C	0.545	0.482	0.471	0.485

*n* number of chromosomes

**Table 4:** Frequency distribution of *CYP2D6* star alleles

Variant	Friedrich et al. [19] (n = 474)	Current study		
		Total (n = 380)	Recurrence (n = 88)	No recurrence (n = 292)
*1	0.418	0.409	0.409	0.456
*2	0.253	0.220	0.148	0.241
*4	0.112	0.086	0.102	0.082
*5	0.027	0.024	0.068	0.010
*9	0.011	0.008	0.00	0.010
*10	0.015	0.050	0.045	0.051
*17	0.055	0.018	0.034	0.014
*29	0.023	0.013	0.000	0.017
*35	0.023	0.00	0.000	0.000
*39	0.015	0.060	0.102	0.048
*41	0.042	0.047	0.068	0.041
Other	0.000	0.024	0.023	0.024
Activity score (phenotype)^b^				
0	–	–	0.023	0.027
0.5	–	–	0.023	0.027
1	–	–	0.295	0.151
1.5	–	–	0.182	0.137
2	–	–	0.341	0.575
> 2	–	–	0.136	0.082

*n* number of chromosomes

Comparison of the two data sets reveals relatively small (< 5% of the total allele count), albeit significant differences (*p* < 0.05, Chi square test) in MAF at the 31G>A, 1023C>T, 1846C>T, 4180G>C and 100C>T SNPs (Table 3). Table 3 shows also the MAF at the nine *CYP2D6* genotyped loci in patients with and without recurrence. Statistically significant deviations from Hardy–Weinberg expectations were not observed at any locus. The variant − 1584G allele (rs1080895) showed lower frequency in patients with recurrence (*p* = 0.015). No other significant differences in MAF were observed.

The distribution of the *CYP2D6* star alleles among *P. vivax* malaria patients and healthy volunteers differed significantly (*p* < 0.001), but the difference in frequency of individual alleles did not exceed 5% of the total allele count. Alleles *10 and *39 were more frequent, whereas *17 was less frequent in the *P. vivax* patients (Table 4). The frequency distribution of the *CYP2D6* star alleles differed significantly between patients with and without recurrences (*p* < 0.001; Table 4). The largest difference was in CYP2D6*2, which is defined by the − 1584G allele and was considerably less frequent in patients with recurrence (*p* = 0.148) than in those without recurrences (0.241). Differences in frequency of other star alleles did not exceed 5% of the total allele count. Regarding AS categories, a non- significant trend (*p* = 0.075) for difference in frequency distribution among patients with and without recurrence was detected.

### *CYP2D6* activity score and *P. vivax* malaria recurrence

For this analysis, *CYP2D6* activity scores (AS) were dichotomized at ≤ 1, comprising gPM, gIM and gNM-S phenotypes, versus ≥ 1.5, comprising gNM-F and gUM phenotypes. Incidence of *P. vivax* recurrences was 32.4% in patients with AS ≤ 1 and 20.9% in patients with AS ≥ 1.5 (crude RR = 1.55, CI 95% 0.89–2.71; *p* = 0.075). After adjusting for age and sex, a significant difference was noted between groups, with higher incidence of recurrence among patients with AS ≤ 1.5, i.e. presenting phenotypes of reduced *CYP2D6* activity (adjusted RR = 1.89, CI 95% 1.01–3.70; *p* = 0.049). AR and PAF were 11.5 and 9.9%, respectively.

**Fig. 1:**
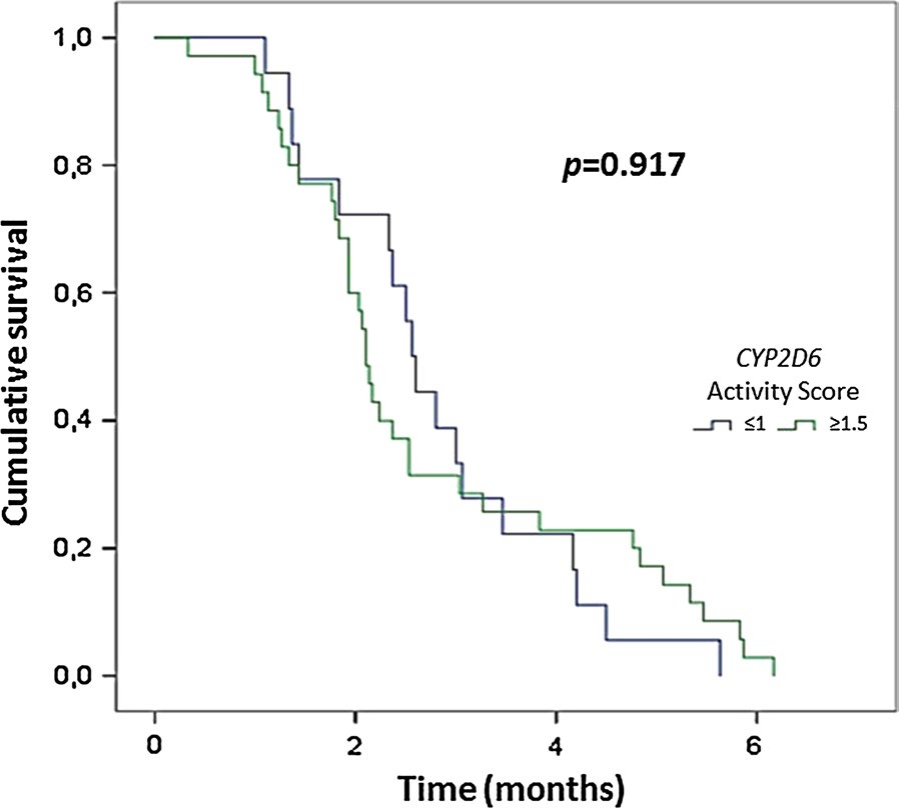
Kaplan–Meier curve for malaria recurrence time between individuals with normal and reduced CYP2D6 activity phenotype. Score ≤ 1 includes poor (gPM), intermediate (gIM) and normal-slow (gNM-S) metabolizers and score ≥ 1.5 includes ultrarapid (gUM) and normal-fast (gNM-F) metabolizers

The time elapsed from the first *P. vivax* malaria episode until the recurrence did not differ significantly between the two groups of patients with AS ≤ 1 or AS ≥ 1.5 (*p* = 0.917; Fig. 1).

## Discussion

The results of the present study point to increased risk of *P. vivax* malaria recurrence in chloroquine/primaquine-treated patients with null (gPM) or reduced (gIM or gNM-S) CYP2D6 metabolic activity compared to gNM-F or gUM metabolizers. This result is consistent with the requirement of *CYP2D6*-dependent conversion of primaquine into redox-active metabolites for hypnozoitical effect and with the original findings and conclusions in the case reports by Bennet and Ingram et al. [10, 11]. The present study shows that *CYP2D6* polymorphisms associate with *P. vivax* recurrence even in a scenario of active transmission, in which reinfections and poor adherence to primaquine regimens are possible [24]. Although recurrences have been associated mostly to subtherapeutic primaquine doses [25, 26], findings presented here show that strategies to achieve radical cure may also be hampered by the presence of poor or intermediate primaquine metabolizers in the population. New therapeutic regimens that do not require *CYP2D6*-mediated drug metabolism may reduce *P. vivax* recurrences in about 10% at the populational level in the field routine according to this finding. As a promising strategy, tafenoquine efficacy in *P. vivax*-infected individuals is not diminished to the same extent as primaquine in *CYP2D6* intermediate metabolizers [14].

Higher relapse frequency in individuals categorized as intermediate metabolizes was also reported by St Jean et al. [14] but it is not clear whether their findings are comparable to this study, since their patients were apparently not genotyped for copy number variation, which is required for inference of CYP2D6 metabolic phenotypes. In another study of *P. vivax* relapse in Brazilians, Silvino et al. compared the frequency of SNPs at five *CYP2D6* polymorphic loci between individuals with single or multiple-relapses. All individuals had a single *CYP2D6* gene copy and the only significant difference between the two groups was higher frequency of the minor 100T allele (rs1065852) among patients with multiple relapses [15]. This allele in combination with other SNP(s) define two *CYP2D6* variants, namely the no function allele *4 and the reduced function allele *10. The study protocol could not distinguish these two alleles nor allowed for inference of the various *CYP2D6* diplotypes known to occur in Brazilians [19].

This study has distinct strengths. First, it covered a considerably larger cohort of patients than previous reports of association of *CYP2D6* polymorphisms and recurrence of *P. vivax* malaria. Second, inference of CYP2D6 phenotypes was based on genotype data from nine polymorphic loci and for gene copy number variation. Third, the frequency distribution of variants at the *CYP2D6* polymorphic loci agreed well with data from healthy individuals from the Amazon region, where the subjects included in this study were recruited. Nevertheless, this study has limitations. Firstly, the prescribed anti-malarial treatment followed the recommendations of the Brazilian Ministry of Health for *P. vivax*, but primaquine administration was not supervised and the possibility of non-adherence to the 2 daily doses of primaquine during 7 days may not be excluded. Also, undisclosed use of non-prescribed drugs or xenobiotics that might affect CYP2D6 function cannot be excluded in this population. Thirdly, individual CYP2D6 phenotype was inferred from genotyping data, according to activity scores of *CYP2D6* diplotypes and there is evidence for considerable range of variation in CYP2D6 function within genotype-inferred phenotype categories.

## Conclusion

In a prospective cohort it was validated that polymorphisms in *CYP2D6* gene associated with increased risk of recurrence of *P. vivax* malaria, following chloroquine–primaquine combined therapy in a field scenario. This association is interpreted as the result of reduced conversion of primaquine into its active metabolites in patients with reduced CYP2D6 enzymatic activity, leading to inappropriate hypnozoite elimination.

## Abbreviations

AR: attributable risk
AS: activity score
CYP: cytochromes P450
CYP2D6: cytochrome P450 2D6
DNA: deoxyribonucleic acid
G6PD: glucose-6-phosphate dehydrogenase
gIM: intermediate metabolizer
gNM-F: normal fast metabolizer
gPM: poor metabolizer
gNM-S: normal slow metabolizer
gUM: ultrarapid metabolizer
MAF: minor allele frequency
PAF: population attributable fraction
PCR: polymerase chain reaction
RR: relative risk
SNP: single nucleotide polymorphism
WHO: World Health Organization
